# Chondroitin Sulfate-Modified Liposomes for Targeted Co-Delivery of Doxorubicin and Retinoic Acid to Suppress Breast Cancer Lung Metastasis

**DOI:** 10.3390/pharmaceutics13030406

**Published:** 2021-03-19

**Authors:** Zhiwei Zhang, Lixin Ma, Jingwen Luo

**Affiliations:** 1 State Key Laboratory of Biocatalysis and Enzyme Engineering, Hubei Key Laboratory of Industrial Biotechnology, Hubei Collaborative Innovation Center for Green Transformation of Bio-Resources, School of Life Sciences, Hubei University, Wuhan 430062, China; zzw@stu.hubu.edu.cn; 2 Key Laboratory of Drug-Targeting and Drug Delivery System of the Education Ministry, Sichuan Engineering Laboratory for Plant-Sourced Drug, Sichuan Research Center for Drug Precision Industrial Technology, West China School of Pharmacy, Sichuan University, Chengdu 610064, China

**Keywords:** chondroitin sulfate, doxorubicin, retinoic acid, liposomes, breast cancer

## Abstract

Breast cancer treatment remains challenging due to high levels of cell metastasis. Chemotherapy drug combinations can inhibit both tumor growth in situ and metastasis to distant organs. Therefore, here, we developed chondroitin sulfate liposomes (CSLs) as a carrier for the co-delivery of retinoic acid (RA) and doxorubicin (DOX) and examined their efficiency in suppressing lung metastasis of breast cancer. CSLs were prepared using CS–deoxycholic acid conjugates and found to encapsulate both RA and DOX via hydrophobic and hydrophilic interactions. The resulting DOX+RA-CSLs were uniformly spherical and showed good serum stability and encapsulation efficiency of 98.7% ± 1.3% for RA and 90.8% ± 2.9% for DOX. Pharmacodynamic experiments in vitro and in vivo also revealed that DOX+RA-CSLs had better anticancer and anti-metastatic activity than CS-free liposomes, single drug-loaded liposomes, and free drug solutions at the same dose (2 mg/kg DOX or RA). Our results suggest that this liposomal delivery system can effectively suppress lung metastasis of breast cancer.

## 1. Introduction

Cancer metastasis, defined as the spread of cancer cells from the primary tumor site to distant tissues or organs, is the leading cause of breast cancer-related death [1,2]. Breast cancer metastases are mainly observed in the lungs, liver, bone, and brain, while more than 60% of breast cancer patients develop lung metastasis at late stages [3]. Although surgery may effectively treat the primary tumor, chemotherapy is necessary to treat late-stage lung metastases [4]. Despite recent advances in chemotherapy, the five-year survival rate of metastatic breast cancer remains low (~20%) and severe side effects occur in many patients, mainly due to off-target toxicity [5,6]. Therefore, novel therapies with high therapeutic efficacy and low systemic toxicities need to be developed to effectively target cancer cell metastasis.

Currently, targeted drug delivery for the inhibition of lung metastasis is mainly based on size-driven systems such as nanoparticles, micelles, and liposomes [7,8,9]. Liposomes are widely used as drug carriers due to their high drug loading capacity and safety [10,11]. The simultaneous presence of a phospholipid bilayer and an inner aqueous core allows the co-encapsulation of lipophilic and hydrophilic drugs, making liposomes an efficient nanoscale drug delivery system [12].

Although such nanocarriers show promising results, their application is still limited due to their poor stability, complex formulation procedures, and potential toxicity [13]. Most available nanocarriers with diameter around 100 nm act through the enhanced permeation and retention effect (EPR) to show at least some therapeutic efficacy in highly vascularized primary tumors [14]. However, small metastatic tumors are usually less vascularized, limiting the access of nanoparticles [15]. In recent years, intratumoral injection has begun to arouse the interest of scholars because of its ability to directly enrich the drug in the tumor [16]. Local delivery of chemotherapy, in combination with immunotherapy, elicits systemic response against cancer and eliminates the challenges of poor drug targeting from intravenous administration [17,18,19]. However, its disadvantage is also obvious, and it is only suitable for superficial tumors rather than deeper tumors. The safety of immunotherapy is also a problem that needs to be solved urgently [20]. Thus, drug delivery systems with well-defined carrier structures, sufficient in vivo stability, and high targeting efficiency are urgently needed to overcome the current drawbacks [21].

Recent studies have reported that the conjugation of mucopolysaccharides such as hyaluronic acid, heparin, and chondroitin sulfate (CS) to nanocarriers can help them deliver tumor-targeting drugs to the lungs, as the relevant receptors are expressed on the surface of highly metastatic breast cancer cells [22,23,24]. These approaches are only beginning to be explored.

Here, we developed a CS-modified liposome-based drug carrier system for the co-delivery of doxorubicin (DOX) and retinoic acid (RA) and explored its ability to inhibit lung metastasis of breast cancer. DOX is a first-line chemotherapeutic drug used to treat several cancers, including breast, ovary, bladder, and lung cancer, as well as breast cancer metastasis [25,26]. DOX can act synergistically with hydrophilic drugs such as RA to halt tumor growth [27]. In particular, RA can inhibit cancer cell proliferation and destroy the Golgi apparatus in cancer cells [28], while showing lower toxicity and higher stability in vivo than other Golgi-disturbing agents [29].

The drug delivery and uptake efficiency of the co-loaded liposomes, hereinafter referred to as DOX+RA-CSLs, were evaluated and compared with the properties of CS-free and single drug-loaded liposomes both in vitro and in vivo. The antitumor effect of DOX+RA-CSLs on breast cancer was also investigated using a murine model. The results of these studies suggest that the co-delivery of DOX and RA using the newly developed multifunctional carrier is a simple and effective approach to inhibiting breast cancer lung metastasis.

## 2. Materials and Methods

### 2.1. Materials

Phospholipids, cholesterol, DOX, and RA were purchased from Meilunbio (Dalian, China). 3-(4,5-Dimethylthiazol-2-yl)-2,5-diphenyl tetrazolium bromide (MTT) and Dulbecco’s Modified Eagle’s Medium (DMEM) were purchased from Sigma-Aldrich (St Louis, MO, USA). Fetal bovine serum (FBS) and RPMI 1640 medium were obtained from Gibco (Grand Island, NY, USA).

### 2.2. Cells and Animals

B16F10 and 4T1 cells were obtained from the Cell Bank of Shanghai, Institute of Biochemistry and Cell Biology, Chinese Academy of Sciences (Shanghai, China). The cells were cultured in DMEM or RPMI 1640 supplemented with 10% FBS at 37 °C in a humidified 5% CO_2_ atmosphere. Female Balb/c mice (20 ± 2 g, 6–8 weeks of age) were purchased from the Experimental Animal Center, Sichuan University. All animal experiments were approved by the Animal Welfare and Ethics Committee of Sichuan University.

### 2.3. Preparation of Drug-Loaded Liposomes

DOX- and/or RA-loaded liposomes were prepared by a thin-film dispersion method [30]. Briefly, the amphiphilic conjugate CS–DOCA, phospholipids, cholesterol, DOX, and RA were dissolved in chloroform at a mass ratio of 5:15:10:1:1. The mixture was then evaporated in vacuo to give a thin lipid film. A 5% glucose solution (1.5 mL) was subsequently added, and the lipid film was incubated in a water bath at 50 °C for 10 min. After hydration, the liposomes were sonicated at 150 W for 5 min (3 s pulse and 2 s pause) and passed through a 0.45-μm filter to afford the desired DOX+RA-CSLs.

### 2.4. Drug Loading Capacity and Loading Rate

In order to determine the drug loading capacity (LC) and encapsulation efficiency (EE) of DOX and RA, drug-loaded liposomes were dissolved in ethanol and sonicated for 10 min to destroy their structure and release the encapsulated drugs. The concentrations of DOX and RA were then determined by high-performance liquid chromatography (HPLC; Agilent 1260, Agilent Technologies Inc., Santa Clara, CA, USA) using an Agilent ODS-C18 column (250 × 4.6 mm, 5 μm) with a mobile phase of 0.1 M sodium acetate aqueous solution (regulated by acetic acid to pH 2.8): acetonitrile: methanol (47:52:1, *v*/*v*) at a flow rate of 1 mL/min. DOX and RA were detected at 254 and 350 nm, respectively, and their LC and EE values were calculated according to Equations (1) and (2):

LC (%) = W_drug loaded_/W_total liposome_ × 100 (1)

EE (%) = W_drug loaded_/W_drug added_ × 100 (2)

where W indicates weight.

### 2.5. Physical Characterization of Liposomes

The particle size, polydispersity index, and ζ potential of the prepared liposomes were measured by the dynamic laser scattering method (Zetasizer Nano ZS-90, Malvern Instruments, Malvern, UK) and their morphology was observed using a transmission electron microscope (TEM, Tecnai 12, Hillsboro, OR, USA). Briefly, the liposomes were diluted and negatively stained with 1% phosphotungstic acid on a copper grid film. Excess liquid was removed using filter paper.

### 2.6. In Vitro Stability of DOX+RA-CSLs

To investigate the stability of DOX+RA-CSLs during short-term storage, they were allowed to stand at room temperature in the presence of 10% FBS, and their particle size was determined by dynamic laser scattering at 2, 4, 6, 8, and 12 h.

### 2.7. Cellular Uptake

The cellular uptake of the drug-loaded liposomes was studied by flow cytometry (BD FACSCelesta, Piscataway, NJ, USA). B16F10 and 4T1 cells were seeded in six-well plates at a density of 1 × 10^5^ cells/well and cultured overnight at 37 °C. The tumor cells were then washed twice with phosphate-buffered saline (PBS) and incubated in 1 mL of a serum-free medium containing a solution of DOX and RA (DOX+RA-S), CS-free DOX/RA co-loaded liposomes (DOX+RA-Ls), or DOX+RA-CSLs (at an equivalent concentration of 1 μg/mL DOX and/or 1 μg/mL RA). After incubation for 2 h, the cells were trypsinized, centrifuged at 1500 rpm for 3 min, collected, and suspended in PBS. The fluorescence intensity of each drug was finally measured using flow cytometry.

### 2.8. Cytotoxicity Assay

The cytotoxicity of DOX-loaded liposomes (DOX-CSLs), RA-loaded liposomes (RA-CSLs), DOX+RA-CSLs, DOX+RA-Ls, DOX+RA-S, and drug-free CSLs (blank) toward B16F10 and 4T1 cancer cells was evaluated using the MTT assay. Specifically, B16F10 or 4T1 cells were seeded in 96-well plates at a density of 1 × 10^4^ cells/well. After incubation for 24 h, the various formulations were separately added into the medium at different concentrations (0–20 μg/mL). After incubation for 12 h, 20 μL of MTT solution was added to the medium followed by incubation for another 4 h. The optical density (OD) of each sample was measured at 450 nm using a microplate reader with the blank culture medium as control. The cell inhibition rate (%) of each liposome sample was calculated according to Equation (3):

Cell inhibition rate (%) = (OD_sample_ − OD_control_)/(OD_normal_ − OD_control_) × 100 (3)

### 2.9. Wound-Healing Assay

The effect of the developed liposomes on cell migration was evaluated using the wound-healing assay. B16F10 and 4T1 cancer cells were seeded into 12-well plates at 5 × 10^4^ mL^−1^ and cultured for 24 h. When the cells grew to 80% confluence, the cell monolayer was scraped with the same sterile 200 μL pipette tip and washed three times with PBS. Growth medium containing DOX-CSLs, RA-CSLs, DOX+RA-CSLs, DOX+RA-Ls, DOX+RA-S, or drug-free CSLs (blank) was then added, and, after incubation for 24 h, the cells were observed under an inverted fluorescence microscope. Dosage was 1 μg/mL DOX or RA.

### 2.10. Lung Metastasis Model and Drug Administration

To establish the lung metastasis model of breast cancer, 4T1 cells (1 × 10^6^ in 100 μL saline) were injected into the lateral tail vein of eight-week-old female Balb/c mice maintained under specific pathogen-free conditions (25 °C; relative humidity 45%). Lung metastatic nodules of breast cancer were observed at seven days post-administration, confirming the establishment of the metastatic model. Mice bearing lung metastatic nodules of breast cancer were divided into six groups (*n* = 4) and injected via the tail vein with DOX-CSLs, RA-CSLs, DOX+RA-CSLs, DOX+RA-Ls, DOX+RA-S, or drug-free CSLs (blank) at an equivalent dose of 2 mg/kg DOX and/or 2 mg/kg RA once every three days for a total of three times. The same volume of saline was used as control. The mice were weighed every three days and the lung metastatic condition was evaluated by weighing the lungs and counting the total number of metastatic nodules in each lung.

### 2.11. Histological Studies

The lungs, heart, intestine, and stomach were excised from the model mice and fixed for slicing. The isolated sections were stained with hematoxylin and eosin (H&E) for histopathological evaluation, or with antibodies against Ki67 and CD31 for immunohistochemical evaluation.

### 2.12. Statistical Analysis

Statistical analysis was performed using a two-tailed Student’s *t*-test for two groups and one-way analysis of variance (ANOVA) for multiple groups. Data were expressed as mean ± standard deviation (SD). Differences associated with * *p* < 0.05 were considered statistically significant.

## 3. Results and Discussion

### 3.1. Drug Encapsulation and Liposome Characteristics

**Figure 1:**
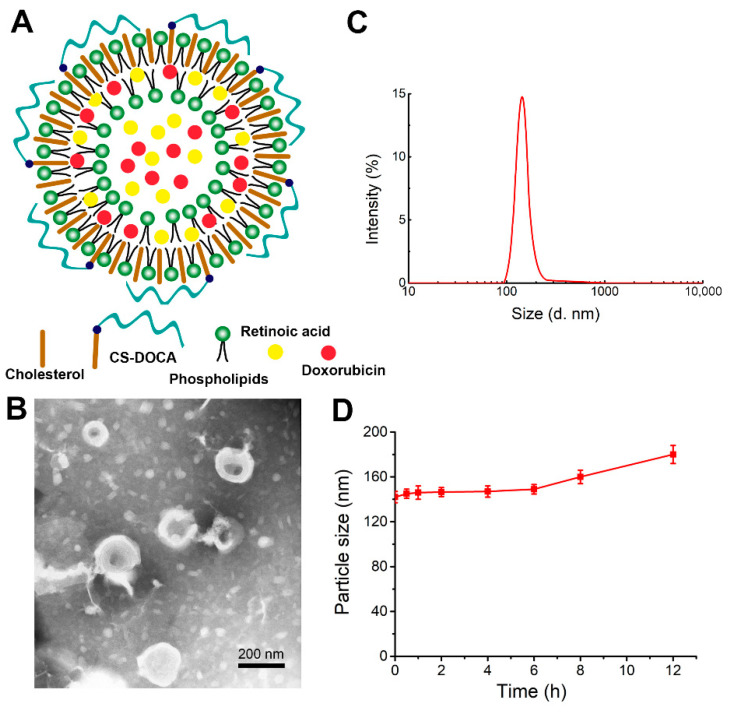
Physical properties of liposomes encapsulating both doxorubicin (DOX) and retinoic acid (RA) (DOX+RA-CSLs). (**A**) hypothesized structure of a single DOX+RA-CSL; (**B**) representative transmission electron micrograph of DOX+RA-CSLs; (**C**) diameter of DOX+RA-CSLs determined by dynamic light scattering; (**D**) stability of DOX+RA-CSL particles in the presence of 10% fetal bovine serum. Data are expressed as mean ± SD (*n* = 3).

The liposomes of this study were prepared from phospholipids, cholesterol, and an amphiphilic polymer of deoxycholic acid (DOCA) attached to CS (CS–DOCA) via an amide bond at an optimal mass ratio of 3:2:1. Using a simple thin-film dispersion method, hydrophobic DOX was then encapsulated via hydrophobic and π–π stacking interactions, while hydrophilic RA was able to co-load due to the inner aqueous core of liposomes (Figure 1A). According to HPLC measurements, DOX+RA-Ls and DOX- and/or RA-loaded CSLs had high EE for both drugs (Table 1), suggesting that the CS coating did not affect the loading efficiency of DOX or RA.

The morphology of DOX+RA-CSLs was observed by TEM (Figure 1B), which clearly indicated their unique phospholipid bilayer structure and suggested that DOX+RA-CSLs were uniformly spherical liposomes with a diameter of ~140 nm, consistent with dynamic light scattering measurements (Figure 1C). The uniform particle size of the prepared liposomes probably resulted from the sonication energy applied during their preparation [31]. In addition, the polydispersity index of DOX+RA-CSLs was lower than 0.20, implying the formation of monodisperse liposomal particles.

The stability of DOX+RA-CSLs was also evaluated in vitro in the presence of 10% (*v*/*v*) FBS at 37 °C, which simulated the body fluid environment. The particle size of DOX+RA-CSLs remained fairly stable for 12 h (Figure 1D), suggesting their sufficient stability as drug delivery nanocarriers in vivo.

### 3.2. Inhibition of Cell Migration In Vitro

**Figure 2:**
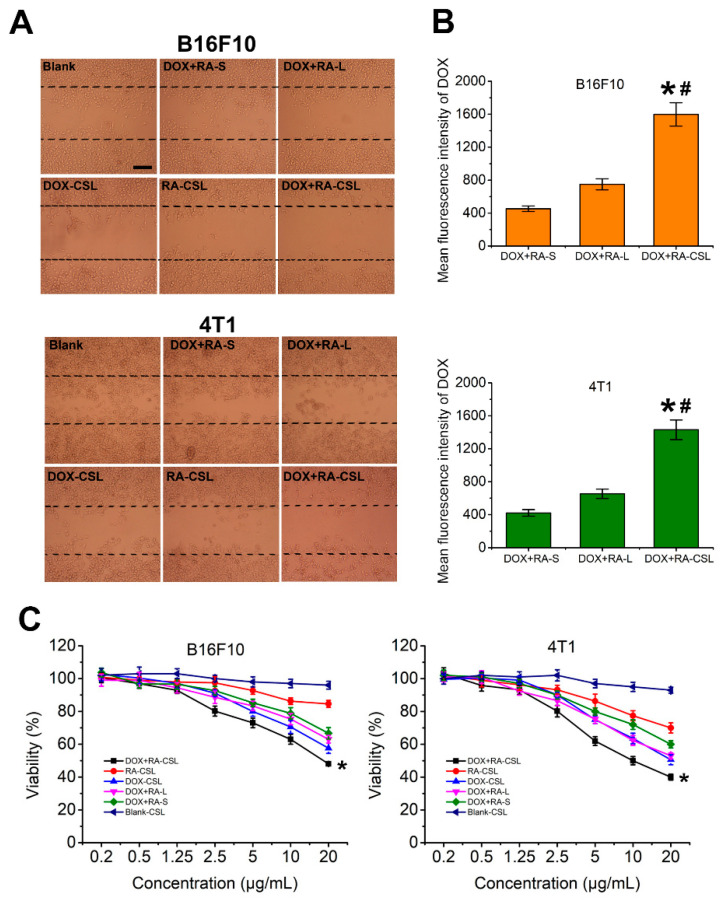
In vitro evaluation of DOX+RA-CSLs. (**A**) inhibition of B16F10 and 4T1 cell migration after treatment with various formulations for 24 h, Bar, 50 μm; (**B**) mean fluorescence intensity of DOX in DOX+RA-S-, DOX+RA-L-, and DOX+RA-CSL-treated B16F10 and 4T1 cells. Data are expressed as mean ± SD (*n* = 3). * *p* < 0.05 versus DOX+RA-S group, ^#^ *p* < 0.05 versus DOX+RA-L group; (**C**) cytotoxicity of drug-free and drug-loaded liposomes toward B16F10 and 4TI cancer cells after incubation for 12 h. Data are expressed as mean ± SD (*n* = 3). * *p* < 0.05 vs. Blank CSL group. CSLs: chondroitin sulfate liposomes; RA-CSLs: retinoic acid-loaded CSLs; DOX-CSLs: doxorubicin-loaded CSLs; DOX+RA-S: DOX/RA solution; DOX+RA-Ls: liposomes co-loaded with DOX and RA; DOX+RA-CSLs: CSLs co-loaded with DOX/RA.

RA is an anti-metastatic drug that interferes with the function of the Golgi apparatus [32]. Given that most proteins related to cancer metastasis are glycosylated on the Golgi apparatus [33], its destruction should inhibit metastasis. Here, we assessed the inhibitory effect of DOX+RA-CSLs by measuring the migration of B16F10 and 4T1 cells in vitro using the wound-healing assay [34] (Figure 2A). The wounded area was substantially repopulated by B16F10 and 4T1 cancer cells after 24 h in all cases except for the DOX+RA-CSL-treated cells, where cell migration was inhibited by 90%. In contrast, DOX+RA-Ls, DOX-CSLs, and RA-CSLs achieved only moderate inhibition (∼20%), which was significantly lower than that of DOX+RA-CSLs but better than that of drug-free CSL- and DOX+RA-S-treated cells. This result may be related to cellular uptake (Figure 2B) and cytotoxicity (Figure 2C). The concentration of the DOX used in the cell migration assay was 1 μg/mL, which, as indicated by the cytotoxicity results, was not cytotoxic. Thus, the cytotoxic effect of DOX can be ignored. Different preparations have different cellular uptake efficiency, which was CS-modified liposomes > liposomes > Drug solutions. RA could destroy Golgi apparatus. Its destruction would inhibit metastasis. Thus, the inhibition of cell migration was DOX+RA-CSL > DOX+RA-L > DOX+RA-S.

### 3.3. Cellular Internalization and Cytotoxicity of DOX+RA-CSLs

The cellular uptake efficiency of the drug-loaded liposomes was quantitatively assessed by determining the concentrations of DOX internalized in B16F10 and 4T1 cell lysates with a flow cytometer. After incubation for 4 h, the mean fluorescence intensity of DOX in DOX+RA-CSL-treated B16F10 and 4T1 cells was significantly higher than that in DOX+RA-L- and DOX+RA-S-treated cells (Figure 2B). For B16F10 cells, the uptake of DOX in DOX+RA-CSLs group was 2.1-fold and 3.5-fold higher than DOX+RA-Ls and DOX+RA-S group. For 4T1 cells, the uptake of DOX in DOX+RA-CSL group was 2.2-fold and 3.4-fold higher than DOX+RA-Ls and DOX+RA-S group, indicating that DOX+RA-CSLs significantly enhanced the cellular uptake of DOX.

Further tests showed that DOX+RA-CSLs were more cytotoxic against B16F10 and 4T1 cells than DOX+RA-Ls or DOX+RA-S at the same dose (Figure 2C). This difference could be attributed to the CS modification, which probably increased the binding affinity of the liposomes for the CD44 receptor on the cancer cell membrane and facilitated drug delivery in breast cancer. The CD44 receptor is overexpressed in various tumor cells, especially in highly metastatic tumor cells [35]. Moreover, CS in proteoglycans is known to participate in the swelling and hydration of the collagen fibril framework, intracellular signaling, and interaction between cells and the extracellular matrix, and has recently been used to mediate drug delivery into CD44-expressing cells [36].

DOX+RA-CSLs were also more cytotoxic than DOX-CSLs and RA-CSLs, indicating the favorable synergistic effect of DOX and RA compared to single drug administration. In contrast, drug-free CSLs showed negligible cytotoxicity against both cell types, allowing nearly 100% cell viability at all concentrations (Figure 2C).

### 3.4. In Vivo Antitumor and Anti-Metastatic Efficacy of DOX+RA-CSLs

The in vivo antitumor efficacy of DOX+RA-CSLs was evaluated in a breast cancer lung metastasis model generated by tail–vein injection of 4T1 tumor cells into female Balb/c mice. Seven days after tumor inoculation, various treatments were injected intravenously to each group of mice (*n* = 4) once every three days for a total of three times. The changes in the lung tumor weight were analyzed five days after the last treatment.

**Figure 3:**
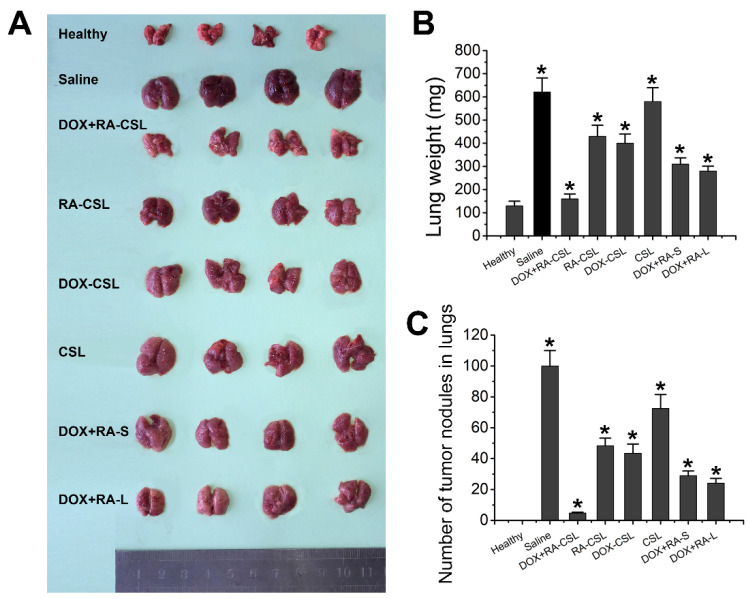
Suppression of lung metastasis of breast cancer in vivo. (**A**) representative gross images of lungs collected from mice receiving various treatments; (**B**) lung weight and (**C**) number of tumor nodules in lungs of mice in different treatment groups. Data are shown as mean ± SD (*n* = 4). * *p* < 0.05 vs. Healthy group CSLs: chondroitin sulfate liposomes; RA-CSLs: retinoic acid-loaded CSLs; DOX-CSLs: doxorubicin-loaded CSLs; DOX+RA-S: DOX/RA solution; DOX+RA-Ls: liposomes co-loaded with DOX and RA; DOX+RA-CSLs: CSLs co-loaded with DOX and RA.

The DOX+RA-CSL group showed a clear therapeutic effect compared to the saline group, while the lungs of this group were similar to the lungs of healthy mice. In contrast, DOX-CSLs, RA-CSLs, DOX+RA-S, and DOX+RA-Ls only moderately inhibited lung metastasis, but we observed a significant reduction in the number of tumor nodules compared to the saline group. Nevertheless, DOX+RA-CSLs were the most effective in inhibiting 4T1 tumor metastasis among all treatment groups (Figure 3A–C).

Due to the complexity of breast cancer metastasis, it is important to develop systems that can automatically detect the target organ for drug delivery. The currently developed CD44-mediated tumor targeted liposomes not only enabled the accumulation of both drugs at the tumor site through the EPR effect and active targeting but were also transported to other organs, especially to the lungs, due to unpredictable metastasis. Therefore, the controlled release property of DOX+RA-CSLs could also alleviate the toxicity of DOX and RA to healthy cells, thus enhancing drug tolerance.

### 3.5. Histological Studies

**Figure 4:**
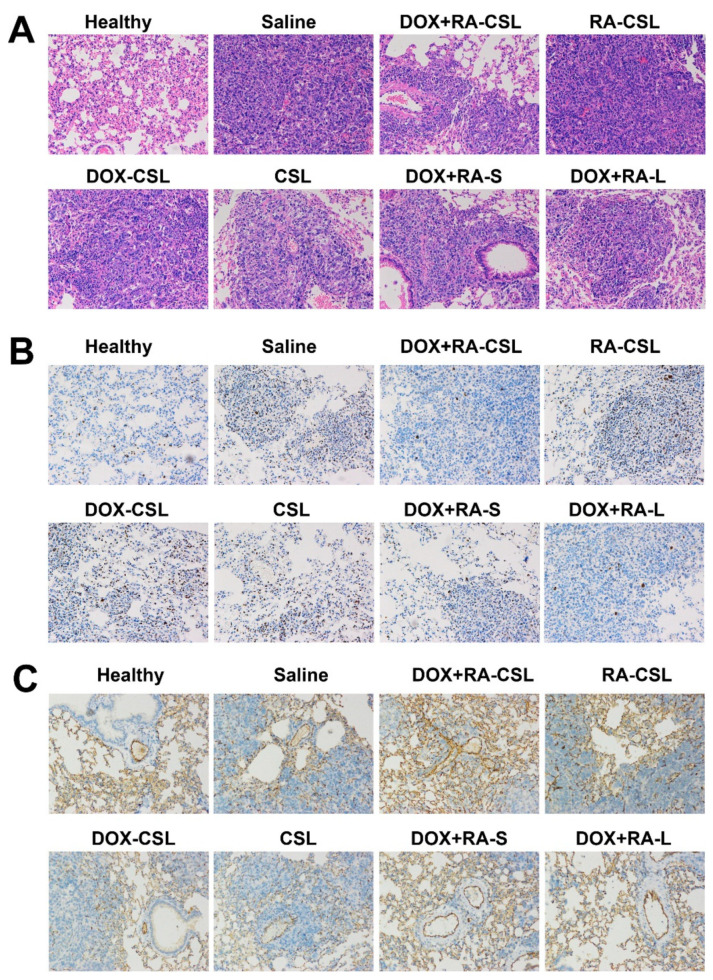
(**A**) H&E, (**B**) anti-Ki67, and (**C**) anti-CD31 staining of lung tissues in mice receiving different treatments. CSLs: chondroitin sulfate liposomes; RA-CSLs: retinoic acid-loaded CSLs; DOX-CSLs: doxorubicin-loaded CSLs; DOX+RA-S: DOX/RA solution; DOX+RA-Ls: liposomes co-loaded with DOX and RA; DOX+RA-CSLs: CSLs co-loaded with DOX and RA.

In order to identify further pathological changes as well as cell proliferation, apoptosis, invasion, and metastasis in mice receiving different treatments, excised lungs from each group of mice were stained with H&E or antibodies against Ki67 or CD31. H&E staining of lung tissues clearly indicated the infiltration of tumor cells (appearing dark with large nuclei) in all treatment groups, while the area of tumor cells increased in the order: healthy < DOX+RA-CSLs < DOX+RA-Ls < DOX+RA-S < DOX-CSLs < RA-CSLs < CSLs < saline (Figure 4A). In addition, the DOX+RA-CSL group had the lowest number of tumor cells.

**Figure 5:**
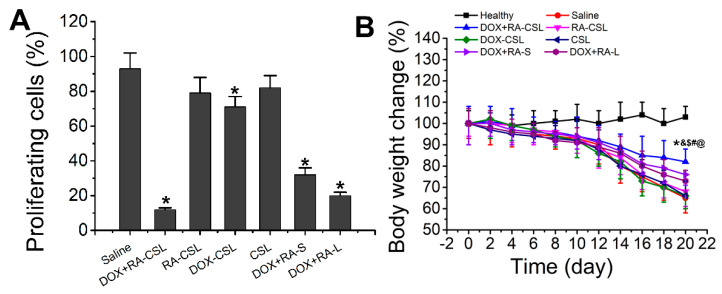
(**A**) Quantitative analysis of Ki67-positive cells in tumor tissues of mice after various treatments; (**B**) temporal change in the body weight of mice receiving different treatments. Data are shown as mean ± SD (*n* = 4). * *p* < 0.05 vs. Saline group, ^&^ *p* < 0.05 vs. DOX+RA-L, ^$^ *p* < 0.05 vs. DOX-CSL, ^#^ *p* < 0.05 vs. RA-CSL, ^@^ *p* < 0.05 vs. Healthy group. CSLs: chondroitin sulfate liposomes; RA-CSLs: retinoic acid-loaded CSLs; DOX-CSLs: doxorubicin-loaded CSLs; DOX+RA-S: DOX/RA solution; DOX+RA-Ls: liposomes co-loaded with DOX and RA; DOX+RA-CSLs: CSLs co-loaded with DOX and RA.

Ki67 is a nuclear antigen associated with tumor cell proliferation and its expression is closely related to the cell cycle [35]. Cells that stain for Ki67 should have strong proliferation ability [37]. Here, the number of Ki67-positive cells in the tumor tissue after treatment with DOX+RA-CSLs was significantly reduced, implying that DOX+RA-CSLs can effectively inhibit tumor cell proliferation (Figure 4B). Moreover, the quantitative analysis of Ki67 staining revealed that DOX+RA-CSLs significantly reduced the percentage of proliferating cells, followed by DOX+RA-Ls, DOX+RA-S, DOX-CSLs, RA-CSLs, CSLs, and saline (Figure 5A).

The density of blood vessels in tumor tissues is associated with metastasis [38]. CD31 is a transmembrane glycoprotein specifically expressed at the junction of vascular endothelial cells in tumor tissues, and its expression level positively correlates with tumor angiogenesis [39]. Anti-CD31 staining of tumor sections from our animals showed that DOX+RA-CSLs significantly inhibited tumor angiogenesis, thus preventing cancer cell metastasis (Figure 4C).

Taken together, the histological results of the DOX+RA-CSL group confirmed the synergistic effect of DOX and RA in the lung metastasis model relative to single drug administration.

### 3.6. In Vivo Toxicity

The in vivo toxicity of the drug-loaded liposomes was evaluated by monitoring the changes in the weight of healthy and tumor-bearing mice throughout the treatment period (20 days). The weight of healthy mice remained almost unchanged, whereas the body weight of all tumor-bearing mice decreased, especially in the saline group, as the lack of treatment probably exacerbated the disease. The smallest weight loss was observed in the DOX+RA-CSL group, indicating that DOX+RA-CSLs had the lowest toxicity and a better therapeutic effect that should favor survival (Figure 5B). The liposome injected mice lost weight compared to the healthy group but gained weight compared to the model group (saline group). Weight loss was common in the diseased mice. DOX+RA-CSL treatment reversed weight loss in mice. It also reflects that DOX+RA-CSL has a good therapeutic effect and low toxicity.

**Figure 6:**
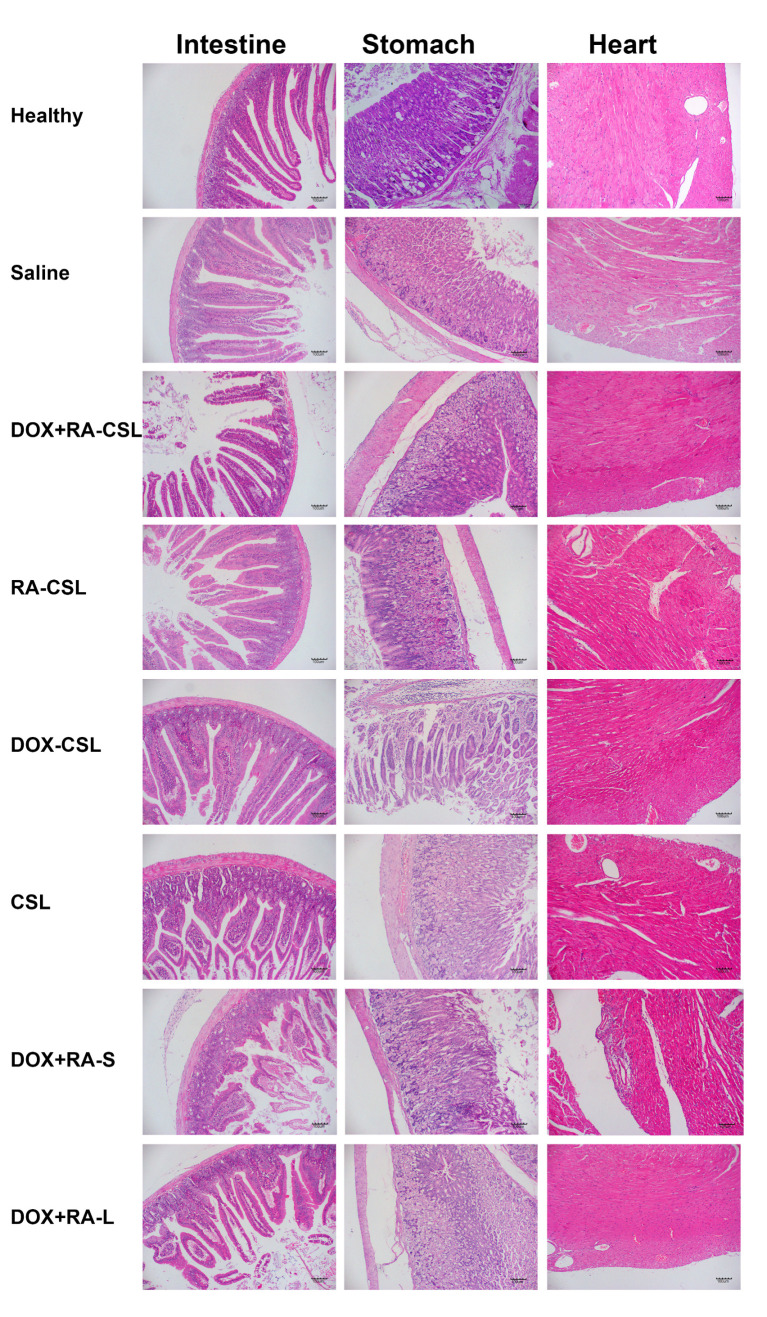
Safety profile of DOX+RA-CSLs determined by H&E staining of intestine, stomach, and heart tissues harvested from mice receiving various treatments. CSLs: chondroitin sulfate liposomes; RA-CSLs: retinoic acid-loaded CSLs; DOX-CSLs: doxorubicin-loaded CSLs; DOX+RA-S: DOX/RA solution; DOX+RA-Ls: liposomes co-loaded with DOX and RA; DOX+RA-CSLs: CSLs co-loaded with DOX and RA.

As a traditional anticancer drug, DOX has cardiac and gastrointestinal toxicity. Therefore, sections of heart, stomach, and intestine from mice receiving various treatments were stained with H&E and analyzed. No considerable difference was observed in the morphology of each organ between the control and DOX+RA-CSL groups except for the DOX+RA-S group, where the cardiac fiber structure was disordered and the myocardial space enlarged (Figure 6). These results suggest that DOX+RA-CSLs may also reduce drug toxicity to non-targeted tissues and organs. Given that phospholipids and cholesterol are biocompatible and biodegradable, and that CS and DOCA are endogenous in the body, we suggest that DOX+RA-CSLs can safely be used for intravenous injection and treatment of lung metastasis of breast cancer.

## 4. Conclusions

In summary, we developed and systematically evaluated the potential of CS-modified liposomes for the targeted co-delivery of DOX and RA with the goal of suppressing lung metastasis of breast cancer. The CS coating significantly improved the cellular uptake and cytotoxicity of DOX+RA-CSLs toward B16F10 and 4T1 cells compared to CS-free liposomes. Further studies showed that DOX+RA-CSLs were stable and hypotoxic in vivo, while they significantly reduced the toxicity of free drugs. The co-loaded liposomes also showed high anti-tumor and anti-metastatic activity in a mouse model of 4T1 lung metastasis, where the co-delivery of DOX and RA led to stronger anti-tumor activity than other treatments. Therefore, we expect that DOX+RA-CSLs will serve as a simple and effective drug delivery system for combination cancer therapy.

## Figures and Tables

**Table 1:** Physical properties of various liposome formulations.

Liposome	Size (nm)	Polydispersity Index	ζ (mV)	Drug Loading Capacity (%)	Encapsulation Efficiency (%)
CSLs	138.5 ± 4.56	0.156	−16.7 ± 0.3	NA	NA
RA-CSLs	139.7 ± 3.54	0.164	−17.1 ± 0.4	3.2	95.3 ± 2.1
DOX-CSLs	142.41 ± 3.23	0.201	−18.2 ± 0.9	3.2	56.2 ± 2.5
DOX+RA-CSLs	141.22 ± 3.41	0.149	−19.5 ± 0.7	6.2	90.8 ± 2.9 ^a^, 98.7 ± 1.3 ^b^
DOX+RA-Ls	137.84 ± 4.09	0.171	−18.5 ± 0.6	6.2	93.4 ± 2.3 ^a^, 97.9 ± 1.6 ^b^

^a^ DOX; ^b^ RA; CSLs: chondroitin sulfate liposomes; RA-CSLs: retinoic acid-loaded CSLs; DOX-CSLs: doxorubicin-loaded CSLs; DOX+RA-CSLs: CSLs co-loaded with DOX and RA; DOX+RA-Ls: liposomes co-loaded with DOX and RA.

## Data Availability

The data used to support the findings of this study are available from the corresponding author upon request.
